# Plausible ergogenic effects of vitamin D on athletic performance and recovery

**DOI:** 10.1186/s12970-015-0093-8

**Published:** 2015-08-19

**Authors:** Dylan T. Dahlquist, Brad P. Dieter, Michael S. Koehle

**Affiliations:** Simon Fraser University, Biomedical Physiology and Kinesiology (BPK), 8888 University Drive – Burnaby, Vancouver, BC V5A 1S6 Canada; UBC Environmental Physiology Laboratory, School of Kinesiology, University of British Columbia, Vancouver, BC Canada; Providence Medical Research Center, Providence Sacred Heart Medical Center and Children’s Hospital, Research Discovery Lab, Spokane, WA 99204 USA

**Keywords:** Vitamin D, Performance, Skeletal muscle, Vitamin K, Dosage, Athlete, Testosterone, Hormones, Recovery

## Abstract

The purpose of this review is to examine vitamin D in the context of sport nutrition and its potential role in optimizing athletic performance. Vitamin D receptors (VDR) and vitamin D response elements (VDREs) are located in almost every tissue within the human body including skeletal muscle. The hormonally-active form of vitamin D, 1,25-dihydroxyvitamin D, has been shown to play critical roles in the human body and regulates over 900 gene variants. Based on the literature presented, it is plausible that vitamin D levels above the normal reference range (up to 100 nmol/L) might increase skeletal muscle function, decrease recovery time from training, increase both force and power production, and increase testosterone production, each of which could potentiate athletic performance. Therefore, maintaining higher levels of vitamin D could prove beneficial for athletic performance. Despite this situation, large portions of athletic populations are vitamin D deficient. Currently, the research is inconclusive with regards to the optimal intake of vitamin D, the specific forms of vitamin D one should ingest, and the distinct nutrient-nutrient interactions of vitamin D with vitamin K that affect arterial calcification and hypervitaminosis. Furthermore, it is possible that dosages exceeding the recommendations for vitamin D (i.e. dosages up to 4000-5000 IU/day), in combination with 50 to 1000 mcg/day of vitamin K_1_ and K_2_ could aid athletic performance. This review will investigate these topics, and specifically their relevance to athletic performance.

## Introduction

Vitamin D, a fat-soluble vitamin, was first discovered in cod liver oil [1] and has since been identified as an essential vitamin, acting as a precursor steroid to a host of metabolic and biological processes. Once converted into its biologically-active form, 1,25-dihydroxyvitamin D [2], it regulates the expression of over 900 gene variants [3]. These gene expressions have been shown to have significant impact on a wide variety of health and performance-related variables, such as exercise-induced inflammation, tumour suppressor genes, neurological function, cardiovascular health, glucose metabolism, bone health and skeletal muscle performance [4–10]. Surprisingly enough, 88.1 % of the world’s population has inadequate vitamin D levels [11]. Deficiency has been shown to be linked to a variety of adverse psychological and health outcomes, such as suicidal thoughts [12], depression [13], cognitive decline and neurological impairment [14], and an increased risk of cancer [15]. Furthermore, individuals with inefficient vitamin D stores have an increased risk of bone disorders like spondyloarthritis [16], rickets [1, 17], and fractures due to higher bone resorption from an overproduction of parathyroid hormone (PTH) [18, 19]. Lastly, deficiency has catabolic effects on muscle tissue [20], causes muscle weakness [21], and impairs cross-bridge formation [22], all of which could impair athletic performance. Due to the increase in enzymatic activity of exercise [23], athletes may be as susceptible, if not more susceptible to becoming vitamin D deficient when compared to the general population. A recent meta-analysis pooling 23 studies with 2313 athletes found that 56 % of athletes had inadequate vitamin D levels [24]. Because of the high prevalence of vitamin D deficiency [25] and its effects on human physiology, this review is aimed to identify the role of vitamin D in athletic performance (for health related aspects of vitamin D, see [11, 18, 26, 27]). This review will cover how vitamin D is metabolized in the body, its potential roles in athletic performance, sources of vitamin D, differences between vitamin D_2_ and vitamin D_3_, optimal levels of vitamin D for athletes, and strategies to achieve these levels and prevent toxicity by nutrient-nutrient interactions.

## Metabolism of vitamin D

**Fig. 1:**
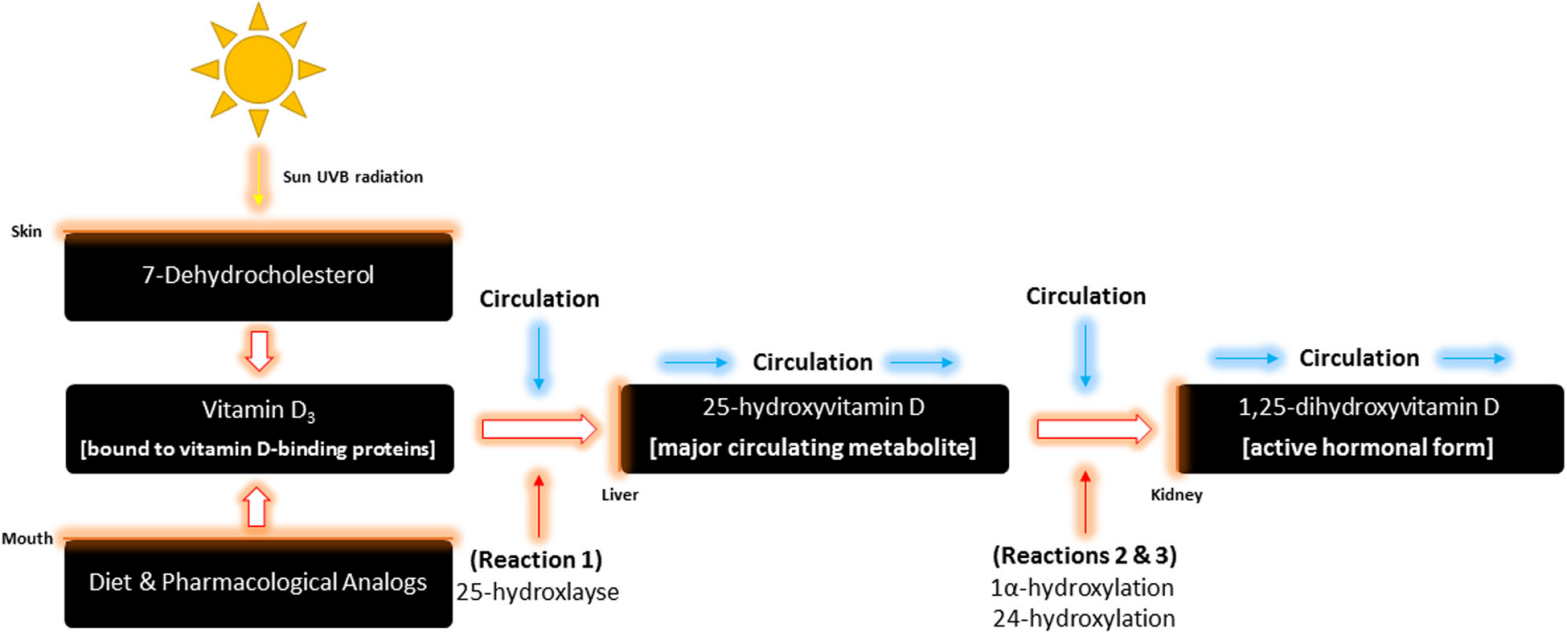
Metabolism of vitamin D_3_ derived from the diet, pharmacological analogs and natural sunlight to the major circulating metabolite of vitamin D (25-hydroxyvitamin D), and subsequently to the active hormonal form, 1,25 dihydroxyvtiamin D

Vitamin D travels in the bloodstream bound to vitamin D-binding proteins [28] and undergoes a three-stage process of key enzymatic reactions [Fig. 1]: 25-hydroxylation, 1α-hydroxylation and 24-hydroxylation [18, 29]. The steroid precursor vitamin D_3_ first travels to the liver where it is hydroxylated to 25-hydroxyvitamin D [25(OH)D] by 25-hydroxlayse, which is mediated by the cytochrome P450 enzymes, CYP27A1 (in the mitochondria) and CYP2R1 [29]. This 25(OH)D is then hydroxylated by CYP27B1 (1α-hydroxylation) [29]. This final step occurs primarily in the kidney [18], but various other tissues, namely skeletal muscle, have also been shown to express CYP24A1 enzymes, where 25(OH)D becomes the active hormonal form, 1,25-dihydroxyvitamin D [29]. 1,25-dihydroxyvitamin D then interacts with vitamin D receptors (VDR), which are located in almost every tissue in the body [30, 31], and is then transcribed into the cell and binds to vitamin D response elements (VDREs) located in DNA [18]. If 1,25-dihydroxyvitmain D does not interact with VDREs, it is further degraded by CYP24A1 (24-hydroxylase) to the inactive form, calcitroic acid [29].

## Vitamin D and performance

Vitamin D_3_ receptors exist in human skeletal muscle tissue [30, 31], indicating that 1,25-dihydroxyvitamin D has a direct effect on skeletal muscle activity. Research on the muscle effects of vitamin D_3_ [32] is limited to diseased populations [20, 33], or healthy untrained adults [34]. Until recently, a few reviews and meta-analyses have shown that increasing serum 25(OH)D levels in a given population have a positive effect on muscle strength, power and mass [33–35] but the only study that examined the effects in athletes [36], had mixed results. In addition, von Hurst and Beck [36] concluded that the optimal intake and serum concentration of 25(OH)D have yet to be identified in the athletic population.

### Maximal oxygen uptake

Vitamin D receptors (VDR) are present in cardiac muscle and vascular tissue [7], indicating that 1,25-dihydroxyvitamin D might influence maximal oxygen uptake (VO_2_max) via the ability to transport and utilize oxygen within the blood to various tissues. Multiple correlative studies showed a positive correlation between VO_2_max and serum 25(OH)D concentration in non-athletes [Table 1] [37–39]. However many confounding variables were not addressed, such as concomitant multivitamin [38] and supplement intake [37, 39]. Studies performed in athletes are conflicting. Koundourakis and his colleagues [40] found that there was a significant correlation between 25(OH)D levels and performance parameters in 67 Caucasian male professional soccer players (age 25.6 ± 6.2). A linear relationship was seen between pre- and post- offseason measurements of 25(OH)D and muscle strength indicated by squat jump (SJ), countermovement jump (CMJ), sprinting ability (10- and 20- m sprint) and VO_2_max [40]. A more recent publication by Fitzgerald et al. [41], concluded that there was no association between 25(OH)D levels and an individual’s VO_2_max in 52 Caucasian competitive ice hockey players [41]. Additionally, the correlation commonly seen between the serum 25(OH)D concentrations and VO_2_max is inversely related to increases in physical activity and training status [39]. Forney and her colleagues [42] recently investigated the association between serum 25(OH)D, VO_2_max and training status in 39 physically-active college students (20 men, 19 women). They showed that the participants with higher (>35 ng-mL-1) serum 25(OH)D levels had a significantly higher VO_2_max (+20 %) than the low (<35 ng-mL-1) serum 25(OH)D group [42]. However, this correlation was limited to males only.

**Table 1:** Vitamin D correlation and intervention studies on Maximal Oxygen Uptake (VO2max)

Author	Reference #	Population	Subjects/Specimens	Type of study	Intervention	Duration	Results
Gregory et al. 2013	[37]	Healthy adults	213 Healthy Male (*N* = 104) and Female (*N* = 109) Adults (44.8 ± 16.4)	Correlation	3 Groups: Deficient (<50 nmol/L, *N* = 16), Insufficient (>50nmoI/L, but < 75 nmol/L, *N* = 57), Sufficient (>75 nmol/L, *N* = 140)	6 Months	Aerobic Fitness Not Affected by 25(OH)D Levels
Mowry, Costello & Heelan 2007	[38]	Mixed females	59 Non-Trained Females (age 16to 24; 19.86 ± 2.13), 55 Caucasian and 4 Asian (V02max of 39.10 ± 7.18 mL/kg/min)	Correlation	Serum 25(OH)D Levels of 46.19 ± 20.14 ng/mL	-	Significant positive association with V02max and 25(OH)D Levels & Significant inverse assocation with body fat and both V02max and 25(OH)D
Ardestani et al. 2011	[39]	Healthy adults	200 Healthy Adults (age 40 ± 14.4), Male (*N* = 92) and Female (*N* = 108) (V02max of 40 ± 9.1 and 30 ± 8.5, respectively)	Correlation	Serum 25(OH)D levels of 34 ± 13.3 ng/mL	-	25(OH)D concentrations are positively (*p* =0.05) related to V02max & Significant Interaction between 25(OH)D and Self- Reported Hours of Moderate to Vigorous Physical Activity (Higher 25(OH)D = Higher Activity)
Koundourakis et al. 2014	[40]	Athletes	67 Caucasian Male Professional Soccer Players (age 25.6 ± 6.2)	Correlation	Performance Testing: Squat Jump (SJ), Countermovement Jump (CMJ), 10 (10 m) and 20 m(20 m)sprint, Maximal Oxygen Uptake (V02max), and anthropometry	6 Weeks (Pre Off- Sea onto Post Off- Season)	Significant correlations between 25(OH)D and ALL performance parameters for both PRE and POST experimental sessions
Fitzgeral et al. 2014	[41]	Athletes	52 Caucasian Competitive Ice Hockey Players (age 20.1 ± 1.5) (V02max 54.6 ± 4.3)	Cross-sectional	Performance Testing: Maximal Oxygen Uptake (V02peak), Max Heart Rate (HR), Peak RER, Total Exercise Time	1 Month Recruiting Phase During Off-Season (May to June)	All Athletes had 25(OH)D Levels <. 65.0 ng/mL, 37.7 % of the Athletes had 25(OH)D levels of < 32 ng/mL & 25(OH)D status was not significantly associated with any parameter measured
Forney et al. 2014	[42]	Active College Students	39 Physically Active College Students (20 Males, 19 Females)	Correlation	25(0H)D Levels of 20.97 ± 1.97 ng/mL (*N* = 20) or 44.15 ± 2.17 ng/mL (*N* = 19)- Primary Outcomes: BMI, % Body Fat, Resting Metabolic Rate, Maximal Oxygen Uptake (V02max), Power Output (Watts), and Muscle Strength	-	Significant positive relationship seen between V02max and 25(OH)D & Significant negative relationship seen between BMI and 25{OH)D
Jastrzebski 2014	[43]	Athletes	14 Elite Lightweight Rowers	Intervention - RCT	6000 IU/day of Vita min D3 vs Placebo in 25(0H)D sufficient athletes (>30 ng/mL)	8 Weeks	Vitamin D vs Placebo: Significant ↑ in V02max (12.1 % and 10.3 %, respectively) and 25(OH)D concentrations by 400 % (~120 ng/mL)

Intervention trials in the athletic population are scarce. To our knowledge, only one exists looking at the effects of vitamin D supplementation on VO_2_max. Jastrzebski [43] performed a single-blinded trial of supplementation with 6000 IU/day of vitamin D_3_ versus a placebo during an 8-week training cycle in 14 elite lightweight rowers with sufficient 25(OH)D concentrations (>30 ng/mL). They demonstrated a significantly increase in VO_2_max (12.1 % and 10.3 %, respectively) and 25(OH)D concentrations by 400 % (~120 ng/mL). The authors concluded that supplementation of vitamin D_3_ during the 8-week training period significantly improved aerobic metabolism in the elite rowers [43]. Further research is needed to test whether an ergogenic effect exists in athletes who are severely deficient in serum 25(OH)D, and if supraphysiological dosages of vitamin D_3_, such as that used by Jastrzebski, have an ergogenic effect in vitamin D replete athletes in other sport disciplines.

The specific mechanism by which increased levels of 25(OH)D affect VO_2_max remains unclear [39], however this phenomenon could be due to the fact that the CYP enzymes that activate vitamin D_3_ into 1,25-dihydroxyvitamin D_3_ have heme-containing proteins [44] and could potentially affect the binding affinity of oxygen to hemoglobin.

### Recovery

The ability to recover rapidly is important for athletes to train at high intensities more frequently. Human skeletal muscle tissue responds to training stimuli and/or tissue damage through remodeling [45–47]. During recovery, 1,25-dihydroxyvitamin D increases the myogenic differentiation and proliferation [48] and down-regulates myostatin, an inhibitory regulator of muscle synthesis of C2C12 myoblasts in culture [49]. Stratos and colleagues [50] showed this marked increase in skeletal muscle regeneration in a crushed soleus muscle (*in vivo*) of 56 male Wistar rats (300 to 325 g body weight), after a supraphysiological dose of ~100,000 IU of vitamin D. They [50] separated rats into a high (332,000 IU/kg) and low (33,200 IU/kg) dose groups and examined recovery response times to the crushed soleus muscle. When compared to the low dose group, the high dose group had a significant attenuation of apoptosis four days post-injury, indicative of an increase in cellular matrix proteins [50]; which is crucial for skeletal tissue repair [51]. This increase in cellular turnover rate led to the enhanced recovery time, an increase in tetanic force production (only 10 % lower than the non-injured limb), and an increase in twitch force when compared to the control group [50]. As murine models display regenerative capacities that exceed those of humans, it is important to note the limitations of extending the aforementioned findings to humans; however, the finding that vitamin D supplementation enhances the recovery in peak isometric force shortly after intense exercise was recently supported in much lower doses in modestly-active humans [52].

In a randomized, double-blind, placebo-controlled study, Barker et al. [52] demonstrated that 4000 IU/day for 35 days of vitamin D in healthy and moderately active adults attenuated the inflammatory biomarkers alanine (ALT) and aspartate (AST) immediately following 10 sets of 10 repetitions of peak isometric force eccentric-concentric jumps. Furthermore, although peak power output decreased in both the groups, the supplementation group only decreased by 6 %, while the placebo group’s power decreased by 32 % immediately post-exercise [52]. This discrepancy persisted at 48 h [52]. Further research examining higher dosages would be warranted to determine if recovery and power output are improved to a greater degree [Table 2].

**Table 2:** Vitamin D *in vitro*, *in vivo* and intervention studies on recovery

Author	Reference#	Population	Subjects/Specimens	Type of Study	Intervention	Duration	Results
Garcia et al. 2013	[48]	Human - Ex Vivo	Human Myoblasts	*In Vitro*	C2C12 Myoblasts treated with 100 nM of 1,25-D3 or Placebo	1, 4, and 10 Days	↑ in Myogenic Differentiation & Proliferation
Garcia et al. 2011	[49]	Human - Ex Vivo	Human Myoblasts	*In Vitro*	C2C12 Myoblasts treated with 100 nM of 1,25- D3 or Placebo	1, 3, 4, 7, and 10 Days	Down-regulation of Myostatin
Stratos et al. 2013	[50]	Rat Model	56 Male Wistar Rats	Intervention - *In Vivo*	High Dose Group: 332,000 Ill/kg Low Dose Group: 33,200 lU/kg- Regeneration of Crushed Soleus Muscle	42 Days	High vs Low: (1) ↓ in Apoptosis (2) ↑ in Cellular Matrix Proteins (3) ↑ Tectonic Force Production (4) Enhanced Recovery
Barker et al. 2013	[52]	Healthy & Active Males	28 Mode rat ly Active (30-min of exercise 3xWeek) Males (Vitamin D Group Age =30 ± 6, *N* = 14); (Placebo Group Age =31 ± 5, *N* = 14)	Intervention - RCT - Placebo + Double Blind	10 sets of 10reps of peak isometric force jumps 4000 IU of Vitamin D3 or Placebo/Day	28 Days	Vitamin D vs Placebo: (1) ↓ ALT and AST(2) Less of a ↓ in peak power output

### Force and power production

Vitamin D_3_ has also been shown to increase force and power output of skeletal muscle tissue [19], perhaps through the sensitization of calcium binding sites on the sarcoplasmic reticulum, leading to an enhanced cross-bridge cycling and muscular contraction [53]. There is further evidence that vitamin D_3_ might also potentially increase both size and number of type II muscle fibers [20, 54, 55]. These findings have only been supported in mobility-limited elderly (≥65 years old) women [55], and have yet to be tested in the athletic population. On the other hand, increases in force and power production have been studied in athletes with positive results during a randomized placebo-controlled study in 10 male professional soccer players [56]. After an 8-week long intervention of either receiving 5000 IU/day of vitamin D_3_ or a placebo, the vitamin D_3_ group had a significant increase in serum 25(OH)D levels and a significant improvement in both their 10-m sprint times and vertical jump when compared to the placebo group [56]. Confounding variables were well-controlled, in that the authors instructed the athletes to maintain current nutritional intake, and excluded any athlete who was taking a multivitamin, vitamin D, fish oil and/or were regular sunbed users or who just returned from a vacation in a sunlight enriched climate. However, other studies have shown no significant benefit of vitamin D supplementation in athletes with moderately deficient or adequate levels [10, 41, 42], indicating that these performance benefits might be limited to individuals with significant vitamin D deficiency [Table 3].

**Table 3:** Vitamin D correlation and intervention studies on force & power production

Author	Reference #	Population	Subjects/Specimens	Type of Study	Intervention	Duration	Results
Ceglia et al. 2013	[55]	Elderly	21 Mobility-Limited Women (age 2 65) with 25{OH)D levels of 225 to 60 nmol/L	Intervention - RCT- Placebo + Double Blind	4000 lU/Day of Vitamin D or Placebo	4 Months	Vitamin D3 supplementation ↑ intramyonuclear VDR concentration by 30 % and increased muscle fiber size by 10 % in older, mobility-limited, vitamin D- insufficient women.
Close et al. 2013	[56]	Athletes	10 Male Professional Soccer Players	Correlation + Intervention - RCT	5000 lU/Day of Vitamin D3 or Placebo	8 Weeks	Vitamin D vs Placebo: (1) ↑ Serum 25 hydroxyvitamin D (2) ↑ in Vertical Jump (3) Faster 10 m sprint times
Close et al. 2013	[10]	Athletes	30 Club-Level Athletes from UK	Intervention - RCT	Three Groups: Placebo, 20,000lU/Week, or 40,000 IU/week of Oral Vitamin D3 (Performance Testing: 1-RM Bench Press, 1-RM Leg Press and Vertical Jump)	12 Weeks	Both 20,000 IU and 40,000 IU of Vitamin D3 ↑ 25(OH)D over > 50 nmol/L, but had no effect on any performance measurement
Fitzgeral et al. 2014	[41]	Athletes	52 Caucasian Competitive Ice Hockey Players (age 20.1 ± 1.5) (V02max 54.6 ± 4.3)	Cross-sectional	Performance Testing: Maximal Oxygen Uptake (V02peak), Max Heart Rate (HR), Peak RER, Total Exercise Time	1 Month Recruiting Phase During Off-Season (May to June)	All Athletes had 25(OH)D Levels £65.0 ng/mL, 37.7 % of the Athletes had 25(OH)D levels of < 32 ng/mL & 25(OH) D status was not significantly associated with any parameter measured
Forney et al. 2014	[42]	Active College Students	39 Phyiscally Active College Students(20 Males, 19 Females)	Correlation	25(0H)D Levels of 20.97 ± 1.97 ng/mL (*N* = 20) or44.15 ± 2.17 ng/mL (*N* = 19)- Primary Outcomes: BMI, % Body Fat, Resting Metabolic Rate, Maximal Oxygen Uptake (V02max), Power Output (Watts), and Muscle Strength	14 Days	Significant positive relationship seen between V02max and 25(OH)D & Significant negative relationship seen between BMI and 25(OH)D

## Vitamin D and testosterone

Testosterone is an endogenous hormone important for muscular adaptations to training. Naturally low testosterone levels in young men have been linked to decreases in protein anabolism, strength, beta-oxidation, and an increase in adipose deposition [57]. Thus, athletes endeavour to optimize natural androgenic production. A recent cross-sectional study done on 2299 older men (62 ± 11 years of age) showed that 25(OH)D levels correlated with testosterone and androgen levels in men [58]. Low testosterone, or hypogonadism, was identified in 18 % of the participants, and these men had significantly lower mean 25(OH)D levels than the rest of the population. Furthermore, only 11.4 % of the sample had sufficient levels of vitamin D.

Additionally a 12-month, double-blind, randomized control trial in 54 non-diabetic males demonstrated that the group receiving 3332 IU/day of vitamin D had a significant increase in circulating 25-hydroxyvitamin D, total testosterone, bioactive testosterone, and free testosterone levels [59]. These findings support the notion that elevating 25(OH)D levels may augment testosterone production in non-diabetic male subjects, which indicates that vitamin D supplementation might have ergogenic potential through the enhancement of endogenous testosterone production. More research is required in order to investigate this potential role of vitamin D and testosterone levels in various study populations [Table 4].

**Table 4:** Vitamin D correlation, *in vivo* and intervention studies testosterone

Author	Reference #	Population	Subjects/specimens	Type of study	Intervention	Duration	Results
Wehr et al. 2010	[53]	Elderly	2,299Caucasian Male Subjects (age S2 ± 11)	Cross-sectional	-	-	Positive correlation seen between 25(OH)D levels and Testosterone and Androgen Levels
Pilz, Frisch & Koertke 2011	[59]	Healthy Males	54 Healthy Overweight Males (age range 20–49)	Intervention - RCT	3332 lU/Day of Vitamin Dor Placebo	12 Months	Significant ↑ in 25(0H)D, Total Testosterone, Bioactive Testosterone and Free Testosterone Positive relationship between higher
Kinuta et al. 2014	[60]	Rat Model	VDR Knockout Mice	Intervention - *In Vivo*	VDR Knockout Mice - Disruption of VDR gene	-	25(OH)D levels and inhibition of gonadal armoatization of testosterone

The specific mechanism of action of 25(OH)D on testosterone in men could potentially be related to two processes: inhibited testosterone aromatization and enhanced androgen binding. Evidence for both of these mechanisms comes from animal models. Specifically, higher 25(OH)D levels inhibit gonadal aromatization of testosterone in VDR knockout mice [60]. Secondly, VDR and vitamin D metabolizing enzymes have been located in human and rat testis and have been shown to enhance the affinity of androgen binding receptors [57, 61, 62]. This effect increases the rate at which androgens can bind to testosterone-producing glands resulting in higher concentrations of steroid hormones, leading to an increase in skeletal muscle hypertrophy, strength and power output [63, 64].

## Sources

### Sunlight

Humans acquire vitamin D from two different sources, endogenous production after sun exposure, or via the diet (from food or supplementation). Unlike the metabolism of dietary vitamin D, the synthesis of vitamin D_3_ by the skin is a non-enzymatic biological process [65]. Once the skin is exposed to the Sun’s ultraviolet B (UVB) radiation, it then converts stored 7-dehydrocholesterol into circulating vitamin D_3_, 25(OH)D [29] and other isomers [66]. The amount of UVB exposure determines the amount and the specific isomers of vitamin D_3_ that will form [66, 67]. The recommended dosage of sunlight exposure during the summer is five to 20 min per day to 5.0 % of exposed skin at a UVB radiation of 290–315 nm [68, 69] two to three times a week [70]. Additionally, it has been shown that 15 min of adequate (290–315 nm) UVB exposure during the summer months in a bathing suit can produce 10,000 to 20,000 IU of vitamin D_3_ [71]. However, multiple factors can affect the rate and synthesis of vitamin D_3_ [Table 5] [25, 66, 72–74].

**Table 5:** Factors affecting the rate and synthesis of endogenously produced vitamin D

Seasonal Variations in UVB Exposure
Living at Latitudes (~32-42° N or S) That Are Further Away From The Equator
Higher Altitudes
Cloudy Climates
Thick Ozone Layers due to Pollution
Darker Skin Pigmentation (higher melanin [natural sun-block] levels)
Higher Adipose Tissue (obesity)
Older Age
Utilization of Sun-block

### Diet

**Fig. 2:**
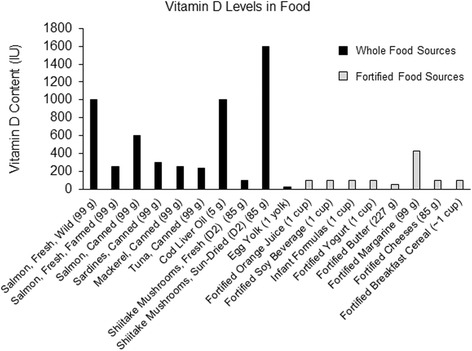
Dietary sources of vitamin D_3_ and D_2_ through whole (natural) or fortified food sources

Vitamin D derived from diet and supplementation comes in two forms, the plant-based vitamin D_2_ (ergocalciferol), and the more bioavailable mammal and fish source, vitamin D_3_ (cholecalciferol) [75]. Vitamin D can be found in various food products [Fig. 2] [76], such as fortified cereals and milk, natural foods like salmon, or through various vitamin D analogues produced synthetically in a laboratory [Table 6] [18, 76]. Both sources are considered prohormone compounds, capable of increasing circulating 25(OH)D, after they have been converted by the enzymatic reactions described earlier.

**Table 6:** Vitamin D sources from pharmacological analogs

Pharmacological supplements	Serving size	Type of vitamin D	Vitamin D (1U)
Prescription			
Pill	1 pill	D2 or D3	50,000
Liquid	1 ml	D2 or D3	8000
Over The Counter			
Multi-Vitamin	1 serv	D2 or D3	1000
400 IU Pill	1 pill	D2 or D3	400
800 IU Pill	1 pill	D2 or D3	800
1000 IU Pill	1 pill	D2 or D3	1000
2000 IU Pill	1 pill	D2 or D3	2000
5000 IU Pill	1 pill	D2 or D3	5000

## Dosage for optimal performance

Both D_2_ and D_3_ are capable of increasing plasma 25(OH)D concentration, but vitamin D_3_ might be more effective than vitamin D_2_ [75, 77, 78]. When compared to vitamin D_3_, vitamin D_2_ is less stable, less bioavailable with increasing age, and it has been shown in multiple clinical studies that the amount of vitamin D_2_ absorbed is significantly lower than with vitamin D_3_. Furthermore, vitamin D_2_ has a lower affinity to VDRs [54, 75, 77–79] and a higher rate of deactivation once hydroxylated in the kidney due to side-chain variations [77]. Lastly, an epidemiological study during the winter months in Dunedin, New Zealand investigated the effects of 1000 IU/day of either vitamin D_2_ or vitamin D_3_ supplementation over a 25-week period in 95 healthy, adult participants (18–50 years old) [78]. The participants who received the vitamin D_2_ supplement had a larger decrease in serum 25(OH)D (74 nmol/L to 50 nmol/L) levels than those who took vitamin D_3_ (80 nmol/L to 72 nmol/L) [78]. However, both results show that 1000 IU/day of vitamin D was inadequate to increase serum 25(OH)D concentrations and actually caused a decline with both isoforms.

With vitamin D_3_ proving more efficacious, the optimal dosage varies depending on the individual and the institution providing the guidelines. The Institute of Medicine (IOM) recommends 400–800 IU/day for children, adults and individuals >70 years of age to maintain serum vitamin D at >50 nmol/L [11, 80, 81]. Alternatively, the Endocrine Society (ES) recommends a slightly higher intake, with dosages of 400–1000 IU/day for infants, 600–1000 IU/day for children, and 1500-2000 IU/day for adults in order to maintain adequate serum vitamin D concentrations of 75 nmol/L [82]. These recommendations correspond with a review in 2004 [83], stating that 70 nmol/L is the lowest desirable serum concentration to prevent adverse health effects. Other recommendations have suggested optimal levels may be 90 to greater than 120 nmol/L [86, 87], based on estimations made from that of levels seen in individuals inhabiting very sunlight-rich environments [84] and/or have shown optimal lower-extremity function [85].

The definitions of *hypo*vitaminosis or *hyper*vitaminosis are more controversial. The IOM defines inadequate stores of 25(OH)D as 30–50 nmol/L, and deficiency as 25(OH)D <30 nmol/L [88], and sets the upper limit of dietary intake of vitamin D to 4000 IU/day [69]. The ES on the other hand, defines vitamin D deficiency at levels of 25(OH)D <50 nmol/L, insufficiency as 25(OH)D between 51 to 74 nmol/L [89], and sets the upper limit of dietary intake of vitamin D to 10,000 IU/day [19]. However, recent reviews have suggested that this is more of a theoretical concern [72, 83, 84, 90]. The optimal vitamin D dosage and level are clearly controversial [88, 91]. Furthermore, the optimal levels needed for athletic performance have not yet been determined. Growing evidence has supported that 600–800 IU/day may not be sufficient for optimal levels of vitamin D, especially for the athletic population [92], since serum 25(OH)D concentrations greater than 100 nmol/L have been proposed to be optimal for lower body skeletal muscle function [85] and low vitamin D levels are linked to increased bone turnover, increasing the risk of stress fractures [93]. It has been shown that it takes roughly 2000 to 5000 IU/day of vitamin D from all available sources in order to optimize bone health by maintaining serum 25(OH)D levels of 75 to 80 nmol/L [84, 85, 94, 95]. Furthermore, this dosage would be unattainable from natural UVB exposure during the months of October to April when residing in latitudes of 42.2 to 52° N [96] which is indicated by the high prevalence of vitamin D deficient indoor and outdoor athletes in a multitude of disciplines [24, 97–99]. Lastly, studies that have shown to improve athletic prowess utilize dosages higher than 3000 IU/day, but none have yet to reach levels greater than 100 nmol/L. Thus, it remains unclear, but athletes may benefit from 25(OH)D levels ≥100 nmol/L in order to increase skeletal muscle function and reduce the risk of stress fractures.

However, no study to date has looked at the effects of vitamin D supplementation and skeletal muscle function in the athletic population with 25(OH)D levels of ≥100 nmol/L [36]. Additionally, the previous performance intervention studies presented in this review supplemented with dosages far greater than the recommended dosages of 600–2000 IU/day (e.g., 5000 IU/day of D_3_) and 1000 IU/day of vitamin D_3_ during the winter months is not enough to prevent a decline in serum 25(OH)D stores [78].

## Toxicity & hypercalcemia

Although it has been reported that vitamin D toxicity might occur with dosages of ≥10,000 IU/day for an extended period [71, 84], producing adverse effects like hypercalcemia, the level of vitamin D causing toxicity is unclear [71, 100], and due to ethical reasons, no prospective studies have analyzed the effect of vitamin D intoxication in humans. Recently, an accidental overdose of 2,000,000 IU of vitamin D_3_ in two elderly patients did not cause adverse effects and only elevated blood calcium levels slightly [101]. More importantly, adverse effects have only been reported at serum concentrations of 25(OH)D above 200 nmol/L, which would take daily dosages of 40,000 IU or more of vitamin D to achieve [84], and serum concentrations of 25(OH)D of <140 nmol/L have not been correlated with hypercalcemia. 1,25-dihydroxyvitamin D works synergistically with calcium and allows it to be absorbed from the gastrointestinal tract and stimulates mature osteoblasts to produce receptor activator nuclear factor-kB ligand (RANKL) [102]. RANKL in turn stimulates mineralization and bone resorption via osteoclastogenesis. Increased levels of 25(OH)D can accelerate this process, causing a rise in calcium concentration in the blood, a higher absorption rate of calcium by the kidneys, and could potentially lead to kidney stones and/or potential vascular calcification [103].

## Vitamin K

Any discussion of vitamin D toxicity merits mention of vitamin K. As with calcium, vitamin K works synergistically with vitamin D to regulate bone resorption, activation and distribution [104]. Vitamin K carboxylates the newly-formed ostecalcin proteins that are produced in mature bone cells and are tightly regulated by vitamin D [105]. Once the protein is carboxylated, it interacts with calcium ions in bone tissue [106] and has a significant effect on bone mineralization, formation, the prevention of bone loss, and potentially the stoppage of fractures in women [105, 107–111]. However, when levels of vitamin K are inadequate, the ostecalcin production is not suppressed [109]. This situation facilitates a build-up of un-carboxylated (inactive) ostecalcin proteins in bone, leading to a potential increase in calcium release from bone and the deposition of calcium into soft tissues (causing arterial calcification) [112, 113]. Thus, vitamin D_3_ toxicity might occur only in the absence of sufficient vitamin K stores.

Recommended dosages of vitamin K range from 50 mcg to 1000 mcg [108]. However, these recommendations are controversial since vitamin K stores are rapidly depleted without constant supply [114] and like vitamin D, vitamin K also has two variants: K_1_ and K_2_. Sources of vitamin K can be found in pharmacological analogues and naturally in the diet. Vitamin K_1_, the most abundant form found in an individual’s diet [115], is high in green leafy cruciferous vegetables, fruits, various vegetable oils and beans [114]. Vitamin K_2_, the more bioavailable form of vitamin K [114], comes in a variety of fish, offal, meat, dairy products, fermented cheese (e.g., blue cheese), and fermented products like natto (fermented soybeans, a Japanese delicacy) [116].

Both forms play different roles in the body [117], but the IOM has set the recommended dietary intake only for the K_1_ isoform (90 mcg/day for women and 120 mcg/day for men), with no upper limit, and has yet to set any dietary recommendation for vitamin K_2_ [114]. Specifically, vitamin K_1_ has a key role in the carboxylation of various blood clotting proteins, where vitamin K_2_ is essential for the carboxylation and activation of osteocalcin and matrix Gla protein (MGP) (an essential protein needed to prevent soft tissue calcification) [118]. More importantly, one of the vitamin K_2_ variants, MK-4, is more effective at mitigating osteoclast formation and the negative health effects of vitamin D overdose [114, 115]. Furthermore, 10 mg/day (10,000 mcg) of synthetic vitamin K_1_ (phytonadione, sold as Konakion® [119]) has been shown to be beneficial for elite female marathon runners by increasing bone formation and preventing bone loss [120] and mega-doses of 45 mg/day (45,000 mcg) of MK-4 in combination with vitamin D_3_ could prevent osteoporosis in postmenopausal women [102, 109]. Thus, although MK-4 might have the greatest effect on carboxylation of osteocalcin, both vitamin K_1_ and K_2_ interact with each other in order to optimize bone health and are essential to the human body. Further research in athletic populations should focus on the optimal dosage for vitamin D_3_ in combination with vitamin K.

## Conclusion

In summary, an interesting theme has emerged from animal studies that supraphysiological dosages of vitamin D_3_ have potential ergogenic effects on the human metabolic system and lead to multiple physiological enhancements. These dosages could increase aerobic capacity, muscle growth, force and power production, and a decreased recovery time from exercise. These dosages could also improve bone density. However, both deficiency (12.5 to 50 nmol/L) and high levels of vitamin D (>125 nmol/L) can have negative side effects, with the potential for an increased mortality [121]. Thus, maintenance of optimal serum levels between 75 to 100 nmol/L [11, 86] and ensuring adequate amounts of other essential nutrients including vitamin K are consumed, is key to health and performance. Coaches, medical practitioners, and athletic personnel should recommend their patients and athletes to have their plasma 25(OH)D measured, in order to determine if supplementation is needed. Based on the research presented on recovery, force and power production, 4000-5000 IU/day of vitamin D_3_ in conjunction with a mixture of 50 mcg/day to 1000 mcg/day of vitamin K_1_ and K_2_ seems to be a safe dose and has the potential to aid athletic performance. Lastly, no study in the athletic population has increased serum 25(OH)D levels past 100 nmol/L, (the optimal range for skeletal muscle function) using doses of 1000 to 5000 IU/day. Thus, future studies should test the physiological effects of higher dosages (5000 IU to 10,000 IU/day or more) of vitamin D_3_ in combination with varying dosages of vitamin K_1_ and vitamin K_2_ in the athletic population to determine optimal dosages needed to maximize performance.
